# Editorial: Advances of Neuroimaging and Data Analysis

**DOI:** 10.3389/fneur.2020.00257

**Published:** 2020-04-08

**Authors:** Jue Zhang, Kun Chen, Di Wang, Fei Gao, Yijia Zheng, Mei Yang

**Affiliations:** Academy for Advanced Interdisciplinary Studies, Peking University, Beijing, China

**Keywords:** neuroimaging, neural mechanisms, fMRI, EEG, deep learning

Neuroimaging is a discipline that studies the structure and function of the nervous system by means of imaging technology, and where the images of the brain can be obtained in a non-invasive way. It explores a series of mechanisms such as cognition, information processing, and brain changes in the pathological state. In recent years, the neuroimaging has developed rapidly and become a powerful tool for medical research and diagnosis. With the increasing prevalence of neurological diseases, higher requirements have been put forward for neuroimaging technology and subsequent data analysis, and many advances have been made in this field.

Now, we will briefly summarize the cutting-edge progress in the theme of “neuroimaging and data analysis.” A total of 16 papers have been published on this topic. They were presented from different countries, including China, USA, Germany, Italy, Brazil, and so on, and they involve novel neuroimaging technology, neuroimaging analysis, clinical diagnosis, and mechanism research. Accordingly, we divide these studies into three sub-topics.

The four papers in the first part of this special issue focused on the practice and development of cerebral hemodynamics in healthy elite athletes and patients, including the exploration of the clinical mechanisms and the discovery of new markers for clinical diagnosis and treatment. Bao et al. reported that by utilizing functional magnetic resonance (fMRI) technology, they realized that fatiguing aerobic exercise changed the cerebral blood supply in the brain and had no significant effect on the ability of the brain to extract oxygenation. Their study provides essential values for the evaluation of anaerobic exercise in sports science and clinics, suggesting that it is meant to establish the CBF and OEF as novel markers for physical and physiological function. Yan et al. assessed the cerebral hemodynamic variations, including bilateral middle cerebral artery (MCA) peak systolic velocity (PSV), pulsatility index (PI), and blood pressure (BP), in unilateral carotid artery stenosis patients with or without Contralateral Carotid Occlusion (CCO) in hours following carotid artery stenting (CAS) using transcranial doppler (TCD) and transcranial doppler color code (TCCD). In particular, they suggested that CCO was a factor of the increased blood flow velocity in ipsilateral MCA after unilateral CAS. Early identification of high-risk patients with transient ischemic attack (TIA) using imaging techniques is essential for administering the proper medications to treat or prevent TIA and the consequent stroke, which will improve the clinical diagnosis of TIA. Thus, Wang et al. explored the probability and related influencing factors of MR Hypoperfusion abnormalities in Chinese patients with transient ischemic attack and normal diffusion-weighted imaging (DWI) findings. Sheng et al. characterized the quantitative DTI-derived diffusion, and DSC-derived perfusion parameter changes underlying different Susceptibility-weighted imaging (SWI) signal intensities of multiple sclerosis (MS) lesions. Moreover, the creatively idea of the work was that the signal intensities detected on SWI in MS lesions might be a non-invasive biomarker that represented a specific stage of lesion evolution or a particular pathological substrate associated with iron deposition, demyelination/axonal injury, or inflammatory activity.

The second subtopic is about the exploration of neural mechanisms of different clinical pathologies and investigations of promising diagnostic methods, including the following eight papers. By using high-field magnetic resonance imaging (7.0-T MRI), Ling et al. analyzed the changes in the lenticulostriate arteries (LSAs) measurements, such as the number of LSA branches and the proportion of discontinuous LSAs in patients with CADASIL. They showed that patients with CADASIL exhibit fewer LSA branches and a higher proportion of discontinuous LSAs than healthy individuals. This suggested that 7.0-T MRI provides a promising and non-invasive method for the study of small artery damage in CADASIL. Song et al. demonstrated the feasibility of performing Functional Ultrasound Imaging (fUS) on two animal models during spinal cord stimulation (SCS). This study could pave the way for future systematic studies to investigate spinal cord functional organization and the mechanisms of spinal cord neuromodulation *in vivo*. Klietz et al. attempted to study the altered brain metabolism in Parkinson's disease (PD) patients systematically with the aid of the whole-brain MR spectroscopic imaging (wbMRSI). They demonstrated that wbMRSI-detectable brain metabolic alterations revealed the potential to serve as biomarkers for early PD.

Generally, how to make better use of image analysis methods to improve the efficiency and accuracy of clinical diagnosis is always an essential issue in neuroimaging. Sun et al. investigated topological organization of the brain structural connectome and demonstrated more severe disruptions of structural connectivity in amnestic mild cognitive impairment (aMCI) converters compared with non-converters. This work may provide potential structural connectome/connectivity-based biomarkers for predicting disease progression in aMCI, which is of great importance for the early diagnosis of Alzheimer's disease (AD). Ruffini et al. proposed a deep learning model for diagnosis/prognosis of Parkinson's disease (PD) derived from only a few minutes of eyes-closed resting electroencephalography data (EEG) and obtained excellent predicting performance, which perhaps contributes a useful tool for the analysis of EEG dynamics. Wuschek et al. aimed to reduce the variance of CSF protein concentrations and, hence, to increase their diagnostic value by considering brain volumes derived from magnetic resonance imaging (MRI). This work can still be considered as a meaningful attempt despite the conclusion that accounting for individual brain volumes is unlikely to decrease the variability of CSF protein concentrations considerably. Moreover, there are also several investigations about the experiment design and pathological characteristics. Schäfer et al. conducted a study to find optimized design paradigms for presenting baby body odors in the fMRI. The paradigms they recommend may transfer to general body odor perception. Jama-António et al. evaluated the frequency of hippocampal atrophy (HA), and the imaging findings and clinical evolution in patients with calcified neurocysticercotic lesions (CNLs), which promotes to identify parenchymal alterations associated with the occurrence of epileptic seizures.

The third subtopic mainly includes four literature reviews, including image analysis methods and challenges of new neuroimaging technology in clinical application. It comprehensively summarizes the functional magnetic resonance imaging, brain structure and aging, and other fields. Franke and Gaser focused on establishing biomarkers of the neuroanatomical aging processes exemplifies for predicting age-associated neurodegenerative diseases. They summarized recent studies that utilize the innovative BrainAGE biomarker to evaluate the effects of interaction of genes, environment, life burden, diseases, or lifetime on individual neuroanatomical aging. Furthermore, they concluded that predictive analysis method could provide a personalized biomarker of brain structure, which helps to clarify and further study the patterns and mechanisms of individual differences in brain structure and disorder stages. Besides the BrainAGE biomarker, simultaneous EEG-fMRI technology could offer the possibility to characterize the relationship between EEG spectrum and regional brain activation, providing new insights on neurological and psychiatric diseases and, hopefully, new treatment targets. Mele et al. paid attention to simultaneous EEG-fMRI technology and related early studies, dealing with issues related to the acquisition and processing of simultaneous signals. They realized that despite this technique appear essential to investigate physiological brain networks in healthy subjects, which introduce new evidence about the electrical neural activity and the neurovascular coupling underpinning the BOLD signal, the optimal integrated and standardized analysis is still open, representing the real challenge that follows the technological development. Moreover, there are many innovative applications based on deep learning in various technical aspects of Neuro-Imaging, particularly applied to image acquisition, risk assessment, segmentation tasks. Zhu et al. addressed this topic and presented an overview. They pointed out that although deep learning techniques in medical imaging have been enthusiastically applied to imaging techniques with many enlightening advances, they are still in the initial stage and face challenges such as overfitting and difficult interpretation of models, lack of high-quality data sets, etc. It is worth mentioning that there is also a very interesting work in this part, O'Connor and Zeffiro summarized the difficulties of resting fMRI (rs-fMRI) in clinical diagnosis thorough investigation, such as availability of robust denoising procedures, and single-subject analysis techniques. The survey results showed that despite some perceived impediments to expanding clinical rs-fMRI use, neuroradiologists were generally confident in the clinical research and application of rs-fMRI.

To sum up, this special issue covers three topics of neuroimaging and data analysis: (1) exploring the physiological mechanism and diagnostic methods of clinical diseases; (2) investigating how the new technology can be effectively applied in clinical practice; (3) tracking the development of cutting-edge technologies. These researches not only contribute to understanding the impact of the development of neuroimaging on the perception of the nerve system, especially in the influence on structure-function and brain-behavior relationships, but also provide new insight into the role of neuroimaging in clinical application. Using imaging techniques to advance the understanding of pathology, abnormal development, and the use of biomarkers or other questions of clinical utility will be an essential part of neuroimaging. However, it is also a problem worthy of attention to objectively view the development of new technology and its proper use in clinical practice. In particular, deep learning, as an excellent and widely used image analysis method, has much work to do to increase its internal interpretability and use limited medical data for practical analysis.

## Author Contributions

JZ organized and proofread the writing of the editorial. KC, YZ, DW, FG, and MY wrote the manuscript draft.

### Conflict of Interest

The authors declare that the research was conducted in the absence of any commercial or financial relationships that could be construed as a potential conflict of interest.

